# Implementation and evaluation of specialist heart failure pharmacist prescribing clinics

**DOI:** 10.1007/s11096-024-01808-9

**Published:** 2024-10-05

**Authors:** Gayle Campbell, Ciara Doherty, Andrew D’Silva, Gerald Carr-White, Jessica Webb, Tevfik F Ismail

**Affiliations:** 1 Cardiology Department, Guy’s and St Thomas’ NHS Foundation Trust, London, SE1 7EH UK; 2 School of BMEIS, King’s College London, London, UK

**Keywords:** Adherence, Heart failure, Pharmacist, Prescribing

## Abstract

**Background:**

Medications form the basis of treatment for heart failure (HF) and adherence is crucial as untreated HF has a mortality of greater than 30%. As such, specialist HF pharmacists with expertise in prescribing and promoting adherence have become an integral part of the wider HF multidisciplinary team (MDT).

**Aim:**

To implement specialist HF pharmacist prescribing clinics and evaluate their impact.

**Setting:**

An integrated HF team at a tertiary London hospital.

**Development:**

The clinic was initially developed to facilitate the introduction of sacubitril-valsartan evolving to 6 dedicated clinics/week.

**Implementation:**

A dedicated electronic referral pathway was created to channel referrals to the specialist clinic, and referral criteria expanded to all patients requiring optimisation of medical therapy.

**Evaluation:**

Data were retrospectively collected for patients with heart failure with reduced ejection fraction seen in the HF pharmacist clinic between September 2021 and July 2022. Overall, 114 patients were seen (mean age 66 years, 78 male). The mean time to medication optimisation was 3 months (averaging 1 appointment/month). The number on optimised doses of guideline-directed medical therapy, increased significantly from 8% at first appointment to 76% on discharge (*p* < 0.001). The HF pharmacists reviewed all medications and optimised non-HF medications for 17.5% (n = 20) of patients.

**Conclusion:**

HF pharmacists can optimise patients’ HF and non-HF medical therapy typically within 3 months. By reviewing all prescribed medications, HF pharmacists provide a holistic review of all medications. They can play a vital role in addressing the underutilisation of HF medical therapy and thereby improving patient outcomes.

## Facilitators to best practice

- Heart failure prescribing pharmacists possess the skills and experience required to optimise guideline-directed medical therapy (GDMT) in patients with heart failure with reduced ejection fraction (HFrEF) but also optimise unrelated pharmacotherapy thereby improving prescribing safety and efficacy.
- As pharmacists have less of a role in diagnostic and prognostic assessment, they have greater clinical availability and scope to focus on implementation of prognosis modifying pharmacotherapy.
- Pharmacists have specific training and skills to assess and promote medication adherence

### Barriers to best practice

- One of the current challenges facing HF teams is the implementation of the growing number of evidence-based medications while supporting patient adherence. HF teams must look to the wider multidisciplinary team to re-design patient pathways to best meet these challenges.
- Skilled HF pharmacists however remain a scarce resource, but this work demonstrates the potential utility of a pharmacist-led approach and provides an impetus for growing the specialist pharmacist workforce.
- Medication optimisation requires multiple patient attendances which may be a barrier to those with limited mobility or financial resources, with potential implications for care equity.

## Background

Globally, the number of newly diagnosed heart failure (HF) patients is increasing, in part due to an expanding and ageing population [1]. This growing burden of HF is placing additional strain on health services [2]. Medications remain the cornerstone for treatment of heart failure with reduced ejection fraction (HFrEF) and when initiated and titrated to the optimised doses [3], have transformed the outlook for patients. Untreated HF has a mortality of greater than 30% [4]. Despite these advances, the improvements in outcomes engendered by modern medical therapy are not being fully realised in clinical practice. Data from the United Kingdom (UK) show that on discharge from hospital, only 56% of patients were on three of the disease modifying drugs for HFrEF [5] (not including the sodium-glucose co-transporter 2 inhibitors). Data for other diverse health systems internationally illustrate that this underutilisation of medical therapy is a global problem [6–8]. It is therefore necessary for HF teams to identify strategies to ensure patients are on an optimal medication regimen and to promote medication adherence.

Pre-existing challenges facing the UK National Health Service (NHS) and other similar health systems have been compounded by the COVID-19 pandemic which has resulted in a backlog of patients who have missed out on routine appointments and made health services less readily accessible. With constrained resources, appointment intervals between HF consultant appointments are typically long. The role of HF nurses and the impact they can have on medicine optimisation is well defined [9]. More recent recommendations have been to include specialist HF pharmacists in the HF multidisciplinary team (MDT) [10].

As medications form the basis of heart failure treatment, this has created an opportunity for the development of specialist HF pharmacists’ roles. There is growing evidence demonstrating the impact pharmacists can have on achieving optimal dosing of quadruple guideline directed medical therapy (GDMT) in HFrEF [11]. Pharmacist interventions have been shown to be associated with a reduction in all-cause mortality and hospitalisations [12]. HF patients tend to have multiple co-morbidities, and HF pharmacists have demonstrated their impact on the review of all medications for safety and optimisation, helping to deliver a holistic review of all medications [13]. However, it is important to understand the type of patients best suited to the HF pharmacist clinics. The different skill-sets of HF pharmacists and nurses, allow an opportunity to ensure the right patient is seen in the right clinic.

### Aim

To implement and evaluate the impact of HF pharmacist prescribing clinics on the uptake of GDMT in patients with HFrEF and review interventions on non-HF medications.

## Development

This was a quality improvement project utilising the Plan-Do-Study-Act (PDSA) methodology [14]. In 2015, a new HF pharmacist specialist post was created at the hospital to work within the HF MDT. Discussions occurred with the wider HF MDT to identify what services the pharmacist would provide, and medication optimisation was identified as a key area. Adherence was also identified an area of concern, given wide variability in rates of adherence has been shown for heart failure patients (40–60%) [15], and it is clear that non-adherence increases the risk of mortality and hospitalisations [16]. Therefore, a HF pharmacist-led medication review and adherence clinic was established, although this was initially non-prescribing. To facilitate referrals, the HF pharmacists implemented medication adherence screening that was completed in the HF nursing clinics for all new patients. This formed the basis for the majority of referrals to the adherence clinic. Where necessary, home visits were also conducted. However, referral numbers were low and patient attendance was poor, in part due to clinic location, but also because it added to appointment burdens. In 2016, a second HF pharmacist post was created and therefore we looked to expand the types of clinics delivered. One of the key considerations in the set-up of the HF pharmacist clinics was how this would fit alongside the HF optimisation already being performed by the HF nurses. A requirement for the HF pharmacists employed is that they are non-medical prescribers (NMPs) and we wanted to utilise this expertise within the clinic setting and combine this with expertise on assessing and promoting medication adherence.

## Implementation

Following the publication of PARADIGM-HF in 2016 [17], sacubitril/valsartan was approved for use by the National Institute of Clinical Excellence in the UK. Local guidelines required the prescriptions to be issued by the hospital until the patient was stable. This created an opportunity to utilise the prescribing ability of the HF pharmacists. The first pharmacist-led prescribing clinics were implemented in 2016, and primarily focussed on converting patients to sacubitril/valsartan. This continued to be the mainstay of referrals, until the COVID-19 pandemic. The pandemic created a significant back-log in HF consultant appointments, and the use of sacubitril/valsartan had become much more frequent. Therefore, prior to recommencing face-to-face consultations in October 2020, the HF pharmacist referral and clinic set-up were reviewed with the key stakeholders.

To support clearing the backlog of consultant appointments and allow them to focus on the management of more complex patients, it was decided to increase the number of patients seen in the HF pharmacist clinics. The increase in capacity occurred alongside amending the referral criteria to accept a broader range of patients. The HF pharmacists would continue to accept patients for conversion to sacubitril/valsartan, but would also now accept patients who were able to or needed to undergo a more rapid optimisation, for example cardio-oncology patients, and those with other forms of heart failure where optimisation was required, for example hypertensive HF. The HF pharmacists previously had a separate clinic to review patients with complex adherence issues or those requiring polypharmacy review, however, with the change in service provision, it was decided to incorporate these patients into the expanded clinics given the synergies involved.

The aim of the HF pharmacist clinic was optimisation of medications, and while the focus was on HF, all medications are reviewed, and interventions made if appropriate. Medicines optimisation is a patient centred approach ensuring the right patient, gets the right medicine, at the right time. It aims to improve medication safety, improve adherence, and reduce waste [18]. A patient is considered optimised if they are on the maximum or maximum tolerated doses of the 4-disease modifying HF medical therapies, with no scope for further up-titration (based on observations, potassium, renal function, side-effects) or if there is a clear contraindication to a therapy. The HF pharmacists used their expertise to undertake HF medicine optimisation, there was no set protocol. However, they worked within the recommendations of locally approved heart failure guidance. The HF pharmacists ensured patients were appropriately counselled on their HF diagnosis, and education provided on this and all medications when needed. Adherence was also assessed and patient specific interventions suggested if non-adherence or factors leading to non-adherence were identified. All eligible patients were also offered referral to cardiac rehabilitation.

Each referral to the HF pharmacist clinic was assessed on an individual basis to ascertain which clinic the patient would be most suitable for, *e.g.*, the HF pharmacist or nursing clinics. The number of clinics sequentially increased, so by July 2021 the team were delivering six clinics weekly, with capacity for 30 patients per week. All HF pharmacist clinic appointments are 45 min long.

The cardiovascular department at St Thomas’ Hospital following the merger with Royal Brompton and Harefield hospitals form part of the largest specialist heart and lung centre in the UK, providing the full spectrum of cardiac and cardiothoracic care, including heart transplantation. The UK National Heart Failure Audit Data show that at St Thomas’ Hospital sees around 600 new unplanned admissions for heart failure per year [5]. HF pharmacists employed at the hospital are required to have completed a postgraduate diploma in general pharmacy practice and have completed their independent prescribing either in heart failure, or within another cardiology sub-speciality. Prior experience managing cardiovascular patients as a ward-based pharmacist is also required. On appointment, an induction period is completed which includes shadowing doctors, nurses, and pharmacists delivering HF clinics. This is followed by delivering a series of supported/supervised HF clinics. The number of supported clinics undertaken is dependent on the ability level of the individual. When the pharmacist is ready, two assessed clinics are undertaken. Only after successful completion of this training, is a pharmacist able to independently deliver HF pharmacist clinics.

## Evaluation

Data were retrospectively collected for patients discharged from the HF pharmacist clinic between September 2021 and July 2022. The hospital patient electronic record was used to obtain demographic data, number of encounters, physiological and laboratory data, total number of medicines, total number of interventions, details on optimisation of the HF medicines, details on non-HF medicine changes, and adherence support. As this was a service evaluation, no ethical approval was needed. However, the evaluation was institutionally approved and registered on the Trust’s audit database. Changes in the rates of prescribing were evaluated with a paired McNemar test with two-tailed values of *P* < 0.05 considered statistically significant.

A total of 120 patients were discharged from HF pharmacist clinic. Of these 114 (95%) had HFrEF, 1 (0.8%) had heart failure with preserved ejection fraction (HFpEF), and 3 (2.5%) had hypertrophic cardiomyopathy (HCM) (Table 1). There were 435 appointment episodes. Most appointments were conducted in-person (317 [73%]), but telephone consultations were used when appropriate on 59 occasions (13.5%). A total of 59 appointments were not attended (13.5%). The average number of clinic appointments per patient prior to discharge from the service was 3. The majority of referrals were from the HF consultant clinics, 111 (93%), with 8 (7%) following hospital discharge, and 1 (1%) referred from a HF nurse. An intervention was defined as a change in dose/medication, addition or cessation of a medicine. If a dose of one medication was increased twice, with a change occurring at differing appointments, this was counted as two interventions. A total of 467 medicine interventions were made by the HF pharmacist across all encounters, an average of 4 per patient (Table 2). Three different HF pharmacists were involved in the delivery of clinics in this evaluation.

**Table 1:** Clinical characteristics and demographics of patients seen within the heart failure pharmacist clinic

Characteristic	n = 120
Mean age ± SD, years	66 ± 13
Male (%)	83 (69)
*Ethnicity*	
White (%)	74 (62)
Black (%)	31 (26)
Asian (%)	10 (8)
*Co-morbidities*	
Ischaemic heart disease (%)	49 (41)
Type II diabetes mellitus (%)	40 (33)
Atrial fibrillation (%)	42 (35)
Hypertension (%)	50 (42)
*Devices*	
Cardiac resynchronisation therapy (%)	26 (22)
ICD (%)	18 (15)
HFrEF only	n = 114
Mean LVEF ± SD, %	30 ± 8

SD = standard deviation; ICD = implantable cardioverter-defibrillator; HFrEF = heart failure with reduced ejection fraction; LVEF = left ventricular ejection fraction

**Table 2:** Summary of referral characteristics, outcomes, and interventions

Characteristic of all patients	n = 120
Total clinic appointments	435
In-person (%)	317 (73)
Telephone (%)	59 (13.5)
Did not attend (%)	59 (12.5)
Average number of appointments per patient	3
*Origin of referral*	
HF consultant (%)	111 (92)
HF nurse (%)	1 (1)
Post-discharge (%)	8 (7)
*Medicines*	
Average number per patient	10
Total number of patients on multi-compartment adherence aids	23
Average number of interventions per patient	4
In HFrEF	114
Average time to optimisation ± SD, months	3 ± 1

HF = heart failure; HFrEF = heart failure with reduced ejection fraction; SD = standard deviation

Adherence was assessed for all patients (100%), utilising the ‘Making Medicines Work for You’ screener [19]. This was developed to support patients and clinicians identify and then discuss adherence issues [19]. Clear non-adherence was identified in 13 patients (11%). Utilising the screener responses, targeted questioning was undertaken to try and identify patient specific issues. Non-adherence can be intentional due to, for instance, patient concerns about medication side effects, and this was identified as the cause for 6 patients. It can also be unintentional, for example, patients forgetting to take their tablets, and this was identified in the remaining 7 patients. Causes for non-adherence can be complex and patient specific. Therefore, all 13 patients underwent targeted counselling to address their individual factors. The most frequent interventions were reminder alarm (5 patients); rationalisation of medicines to reduce the pill burden (4 patients); a change in medication due to experienced side-effects (4 patients); and the use of written medication charts (2 patients). Adherence was re-assessed via self-report at follow-up clinic appointments, with reported improvements in adherence noted across all patients.

The HF pharmacists reviewed all medications and optimised non-HF medications for 17.5% (n = 20) of patients, with 32 individual medication changes made. Most recommendations were in relation to optimisation of other cardiovascular medications, including de-prescribing, with interventions on anti-hypertension medications (n = 11) being the most frequent. Most patients seen in the HF pharmacist clinic had HFrEF, 114 (95%). The mean time to medication optimisation was 3 months (averaging 1 appointment per month).

The number of patients on optimised doses of GDMT increased significantly from first appointment 9 (8%) to discharge 87 (76%). Angiotensin converting enzyme inhibitor (ACEi), angiotensin II receptor blocker (ARB) or angiotensin II receptor-neprilysin inhibitor (ARNI) increased from 25 (22%) to 105 (92%) (*p* < 0.001); beta blocker increased from 82 (72%) to 105 (92%) (*p* < 0.001); mineralocorticoid receptor antagonists (MRA) increased from 61 (54%) to 100 (88%) (*p* < 0.001) and sodium/glucose cotransporter 2 inhibitors (SGLT2i) increased from76 (67%) to 107 (94%) (*p* < 0.001) (Table 3). During pharmacist consultations, 47% of patients were switched from ACEi/ARB to an ARNI (n = 54); these patients were not categorised as optimised at their first appointment. Patients were converted to an ARNI who were symptomatic despite treatment with ACEi/ARB as per current UK national guidance [20].

**Table 3:** Breakdown of changes in rates of medication prescribing before and after optimisation for all 4 agents used to treat HFrEF

N = 114	Optimised dose of HFrEF medicines			Reason if not optimised at discharge		Breakdown of optimisation at discharge^†^		
Medication	At baseline (%)	At discharge (%)	P value	Optimised in another clinic (%)	Patient preference (%)	Max dose (%)	Max tolerated dose (%)	Not tolerated (%)
ACE-I/ARNI/ARB*, n (%)	25 (22)	105 (92)	< 0.001	5 (4.5)	4 (3.5)	68 (65)	36 (34)	1 (1)
Beta-blocker, n (%)	82 (72)	105 (92)	< 0.001	6 (5)	3 (3)	49 (47)	52 (49)	4 (4)
SGLT2i, n (%)	76 (67)	107 (94)	< 0.001	6 (5)	1 (1)	99 (93)	0 (0)	8 (7)
MRA, n (%)	61 (54)	100 (88)	< 0.001	12 (11)	2 (2)	19 (19)	64 (64)	17 (17)

^*^Patients switched to an ARNI were categorised as not optimised at baseline

^†^Maximum dose refers to the maximum licensed dose for heart failure. Maximum tolerated dose refers to the numbers who were on maximum tolerated doses with no scope for further titration where this was below the maximum licensed dose

ACE-I = angiotensin converting enzyme inhibitor; ARNI = angiotensin receptor-neprilysin inhibitor; ARB = angiotensin receptor blocker; SGLT2-I = sodium/glucose cotransporter 2 inhibitors; MRA = mineralocorticoid receptor antagonists; HFrEF = heart failure with reduced ejection fraction

Those who did not achieve optimisation of GDMT before discharge were due to patient preference to not titrate further (n = 10); intolerance of the therapy class (n = 30), or optimisation was achieved in a different parallel HF clinic (n = 29) (Table 3).

At the first appointment, 45 (39%) of patients were on regular loop diuretics, and 9 (8%) using them when required. At discharge, regular use had decreased to 28 (25%), with when required usage increasing to 25 (22%). Of those that had been on a loop diuretic, either regularly or when required at admission (n = 54), the dose was reduced in 28 (52%) patients. Only one patient was on a thiazide diuretic (metolazone) at both admission and discharge. A small number of patients were taking ivabradine at admission, 5 (4%). Ivabradine was continued in these patients, with one patient newly initiated. There were 6 (5%) patients on a combination of hydralazine/isosorbide mononitrate at admission. One patient required cessation due to adverse effects, leaving 5 (4%) on the combination at discharge.

At the time of review in November 2022, 5% of patients had died of all causes (n = 6). It was not possible to ascertain the cause of death for those recorded. No data were collated on re-admissions, as only the hospital patient electronic record system was reviewed, which would not account for admissions to other hospitals.

## Discussion

Our data demonstrate that increased utilisation of pharmacist-led HF medicines optimisation clinics may provide a cogent response to bridging the gap between increasing healthcare need and constrained healthcare resources. Patients were rapidly optimised and adherence issues identified and addressed with tailored interventions. There was a significant increase in the proportion of patients on optimal tolerated GDMT and where appropriate, non-HF medications were appropriately rationalised. Loop diuretic requirements decreased significantly as a result.

There is growing international evidence for the impact HF pharmacists can have on the optimisation of GDMT, thereby contributing to improved quality of life, and a reduction in hospitalisation and mortality [11, 21]. US-based studies have similarly noted that integrating HF pharmacists can help facilitate timely review, particularly in departments with constrained resources [22]. While we did not formally evaluate the impact on medication adherence, our review showed a self-reported trend towards improvement, which matches other published data [23]. However, there is still a need to identify the type of patient’s HF pharmacists are best placed to review. From our experience, the role of the HF pharmacist is complementary to the HF nursing role. The key differences are that HF pharmacists will review all medications prescribed including herbal and non-cardiac medications. HF nurses in general have a more restricted scope of practice in relation to medication review [9]. Our HF pharmacist clinics, have a particular focus on medication adherence. The ability to deprescribe across therapeutic areas may help to promote trust and in turn adherence by reducing medication regimen complexity [24]. As our patient cohort is younger, this allows optimisation to be undertaken relatively quickly (3 months).

The publication of STRONG-HF has demonstrated the safety and efficacy of rapid-optimisation of GDMT post-hospitalisation [25]. For patients in the high intensity care group, there was an 8.1% absolute risk reduction in 180-day all cause death and HF readmissions, and improved quality of life compared with usual care [25]. This data provides even greater impetus to improve not just the rates of utilisation of GDMT, but the speed at which this is achieved. Comparison of age and observational data from the patient demographics from our local review showed a similar patient population to that of STRONG-HF. In addition, patients seen in the HF pharmacist clinics were being relatively quickly optimised (3 months), although this was slow in comparison to the 6 weeks rapid optimisation in STRONG-HF. Nevertheless, our approach suggests that HF pharmacists could play a key role in delivering rapid optimisation protocols.

Our evaluation showed that most referrals to the HF pharmacist clinic were for male patients (69%). It is not clear whether this represents our local population, or bias with who is referred to the pharmacist clinic. Further work needs to be undertaken to understand this difference to ensure we are delivering equitable access to specialist care. However, this gender bias is not unique to our data. Cardiovascular disease is the leading cause of death in women worldwide, however, in comparison to men, women are more likely to be underdiagnosed and less likely to be medically optimised [26]. Women are also underrepresented in HF research, and account for only 20–30% of patients within HF clinical trials [27]. In the development of services, HF teams need to understand this bias in their local population and look to implement recommendations to address this [26].

With the change in management of HF patients, teams need to evaluate their models of service provision. HF teams not only need to consider how to optimise medications, but how to structure the clinics to best utilise the wider HF MDT. The co-location of HF MDT clinics could also be considered to streamline patient pathways. In a time of constrained resources, it could be that HF teams utilise existing patient touch points to facilitate optimisation, such as cardiovascular rehabilitation appointments. Remote reviews where appropriate could also be utilised alongside in-person appointments to circumvent issues such as availability of clinic space. Further HF consultant follow up can then be scheduled on completion of optimisation, unless earlier review is medically indicated. Restructuring the delivery of outpatient clinics has the potential to reduce unnecessary HF consultant appointments and allow them to focus on diagnosis and the management of the most complex patients.

Our HF pharmacist team have become integral with the wider HF MDT and continue to expand and develop the role to help support and improve the delivery of patient-centred holistic care. This advanced HF pharmacist role, is a departure from the more traditional pharmaceutical care-based model [28]. However, by pushing the boundaries, this has created a gap between what would have previously been perceived as an entry level senior job with the current skill-set required to deliver this expanded role. This has highlighted both a need for a formal training programme to support development of future HF pharmacist, which does not currently exist in the UK [29]. However, it has also shown the need to adapt the structure of the HF pharmacist team to ensure there is the opportunity for experienced pharmacists to progress into higher roles (e.g., pharmacist consultant). In the short term, the onus to develop future HF pharmacists will fall on individual hospitals. To meet this need, we are looking to create a HF pharmacist training role. This will allow early career pharmacists to develop the skills and knowledge required to be able to transition to senior HF pharmacist roles. However, to create a sustainable HF pharmacist workforce, policy work at a nation level is necessary.

This evaluation has a number of limitations. First, data was retrospectively collected. As such, certain information was not always available. Secondly, at the point of discharge, not all patients had undergone a repeat echocardiogram, so comparison of left ventricular ejection fraction following medicine optimisation was not possible; however, decreased diuretic utilisation suggests significant symptomatic improvement which may be a surrogate for improved physiology.

## Conclusion

HF pharmacists can optimise patients’ GDMT typically in just 3 outpatient consultations over 3 months. By reviewing all prescribed medications, and supporting adherence, HF pharmacists promote safe and effective medication use. HF pharmacists could play a vital role in addressing the underutilisation of HF medical therapy, and thereby improving patient outcomes.
